# DEVELOPMENT AND VALIDATION OF A NEW LAPAROSCOPIC ENDOTRAINER FOR NEONATAL SURGERY AND REDUCED SPACES

**DOI:** 10.1590/0102-672020200004e1559

**Published:** 2021-01-25

**Authors:** Alberto TORRES, Martín INZUNZA, Cristián JARRY, Francisco SERRANO, Julián VARAS, Alejandro ZAVALA

**Affiliations:** 1 Pediatric Surgery Section, School of Medicine, Pontificia Universidad Católica de Chile, Santiago, Chile; 2 Experimental Surgery and Simulation Center, School of Medicine, Pontificia Universidad Católica de Chile, Santiago, Chile; 3 Department of Digestive Surgery, School of Medicine, Pontificia Universidad Católica de Chile, Santiago, Chile

**Keywords:** Pediatric simulator, Neonatal simulator, Basic simulator, Simulation, Surgical education, Simulador pediátrico, Simulador neonatal, Simulador básico, Simulação, Educação cirúrgica

## Abstract

**Background:**

Pediatric procedures have the difficulty of being performed in reduced spaces. Training in reduced spaces has proven to be different in complexity compared to adult laparoscopic endotrainers.

**Aim:**

To develop and validate a new neonatal/reduced-space endotrainer.

**Methods:**

The simulator was tested and assessed by users with different skill levels and experience in laparoscopic pediatric surgery through an 8-item questionnaire. Construct validity was determined by evaluating the performance of each subject on nine exercises.

**Results:**

A 10.5 x 10 x 18 cm acrylic simulator was created, with an internal working surface of 9 x 9 cm. An HD camera was incorporated, with a 0-180° range of movement. All exercises of a Basic Laparoscopic Training Program were adapted on a scale of 1:0.5 to fit in. From 49 participants, 42 (85.71%) answered the survey; 80.5% considered that the simulator reproduces similar conditions to procedures performed in children under one year of age; 61.1% thought that the simulator represents a difficulty identical to procedures performed in newborns; 73.7% considered that the neonatal simulator is more complicated than the adult simulator. Experts showed significantly better performance in all proposed exercises.

**Conclusion:**

The simulator has a high-quality image and design that allows training with basic tasks. The endotrainer permitted to discriminate between these different skill levels and was well evaluated by users with diverse surgical experience.

## Graphical abstract

[Fig f1a]

**Figure f1a:**
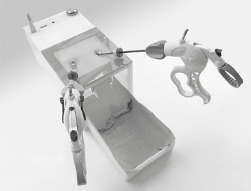
Pediatric laparoscopy box with sliding tray

[Fig f1as]

**Figure f1as:**
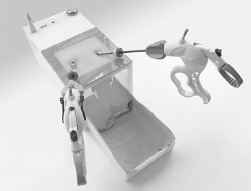
Caixa laparoscópica pediátrica com bandeja deslizante

## RESUMO

**Racional:**

Os procedimentos pediátricos têm dificuldade de serem realizados em espaços reduzidos. O treinamento nesses espaços provou ser diferente em dificuldade em comparação aos endotrainers laparoscópicos adultos.

**Objetivo:**

Desenvolver e validar um novo endotrainer neonatal com espaço reduzido.

**Métodos:**

O simulador foi criado, testado e avaliado por usuários com diferentes níveis de habilidade e experiência em cirurgia pediátrica laparoscópica por meio de um questionário de oito itens. A validação do método foi determinada pela avaliação do desempenho de cada participante em nove exercícios.

**Resultados:**

Foi criado um simulador acrílico de 10,5 x 10 x 18 cm, com uma superfície de trabalho interna de 9 x 9 cm. Uma câmera HD foi incorporada com faixa de movimento de 0-180°. Todos os exercícios do Programa de Treinamento Laparoscópico Básico foram adaptados em escala de 1:0,5 para se ajustarem. Dos 49 participantes, 42 (85,71%) responderam à pesquisa; 80,5% consideraram que o simulador reproduz condições semelhantes às de procedimentos realizados em crianças menores de um ano; 61,1% consideraram que o simulador representa dificuldade semelhante aos procedimentos realizados em recém-nascidos; 73,7% consideraram que o simulador neonatal é mais difícil que o simulador adulto. Especialistas apresentaram desempenho significativamente melhor em todos os exercícios propostos.

**Conclusão:**

O simulador possui imagem de alta qualidade e design que permitem o treinamento com exercícios básicos. O aparelho permitiu discriminar entre os diferentes níveis de habilidade e foi bem avaliado por usuários com experiência cirúrgica diversificada.

## INTRODUCTION

Simulation has proven to be a fundamental tool in surgical education^2^ ^,^ ^8^ ^,^ ^9^ ^,^ ^19^ ^,^ ^20^ ^,^ ^22^ ^,^ ^30^. In recent years we have seen how the design and use of different surgical models have demonstrated to shorten learning curves^9^ ^,^ ^21^ ^,^ ^30^. Surgeons and residents can learn surgical techniques with the possibility of making mistakes and practice abilities and procedures in standardized and supervised situations^1^ ^,^ ^13^. Also, simulation has been incorporated in the resident selection and academic evaluations^7^ ^,^ ^15^ ^,^ ^28^. In 1990 the Society of American Gastrointestinal and Endoscopic Surgeons (SAGES) formed a committee to develop educational material focused on the fundamental aspects of laparoscopic surgery. In 2004, the program of education and evaluation of the fundamental essential elements of laparoscopic surgery (FLS) was published. FLS’ practical module, based on the McGill models (MISTELS)^10^, describes seven exercises to assess surgical skills. Only five of them demonstrated the ability to discriminate between novice and expert subjects; these were: transfer of objects, cut of figures, use of an endoloop, intracorporeal suture and extracorporeal suture knot^10^ ^,^ ^26^. Our center has actively developed laparoscopic simulation research and education, both in the training of essential skills and advanced surgical procedures, based on previously validated programs and also developing local programs^12^ ^,^ ^16^ ^,^ ^27^ ^,^ ^30^. Pediatric and neonatal surgery have the difficulty of working in small spaces compared to the adult patient dimensions. Many training models have been described and validated for pediatric laparoscopic surgery, frequently using animal models such as mice, rabbits, or pigs^4^ ^,^ ^5^ ^,^ ^6^ ^,^ ^11^ ^,^ ^25^ ^,^ ^29^. However, logistical difficulties are higher, thus implying higher costs and ethical objections, making them difficult to be justified today. In 2011, Azzie et al.^3,^ developed a simulator with pediatric dimensions, validating the basic laparoscopic skills exercises described in the FLS. However, other models of surgical simulation derived from the one described by Azzie et al^.3^ have not been described to our acknowledgment; neither its use by other groups. 

The objective of this study was to present and validate a new training box that allows us to reproduce reduced space conditions and simulate neonatal surgical situations to carry out systematic and standardized training in basic and advanced simulation programs.

## METHODS

No informed consent nor IRB approval was required for this study. 

### Reduced space training box

We describe the main specifications of the reduced space/neonatal training box and image features.

### Reduced Space Basic Laparoscopic Training Program

The Basic Skills Laparoscopic Training Program of our institution is the first step of the whole Laparoscopic Training Program of the General Surgery Residency. This program was developed in the Experimental Surgery and Simulation Center of the School of Medicine of the Pontificia Universidad Católica de Chile, based in FLS and MidWestern programs^10^ ^,^ ^16^ ^,^ ^26^ ^,^ ^27^. Since 2010, all first-year residents from our general surgery residency program must complete this first step. Only then, they can do other laparoscopy programs and pass through the rest of the residency. Our Basic Skills Laparoscopic Training Program is composed of 10 exercises from lower to higher complexity. We modified the size (1:0,5 ratio) of the exercises of this basic program to fit the reduced space of the newly designed neonatal laparoscopy training box. 

### Subjects of study

Pediatric surgeons, pediatric and general surgery residents, and medical clerks forming three groups, were invited to perform nine basic exercises on the neonatal endotrainer. We defined “Expert” as those who had already received simulated laparoscopic training AND performed most of their procedures laparoscopically (>50% of weekly surgeries performed laparoscopically).

### Construct validity

This level of validation allows determining if a specific exercise or task, performed under the experimental conditions, can accurately discriminate between different levels of expertise. Nine basic exercises were taught to every participant. Then, they were asked to perform three times each task (without previous practicing), and the best time was recorded. An exception to this was the intracorporeal knot skill, which was assessed only once. The performance was measured in seconds.

### Survey to surgeons and residents

**FIGURE 1:**
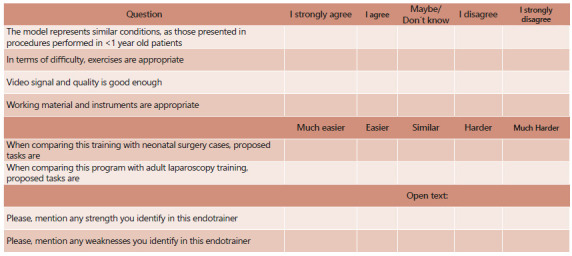
The 8-item questionnaire designed to assess the properties of the simulator concerning its similarity with procedures in reduced spaces

An 8-item questionnaire (Figure 1) was designed to assess the properties of the simulator concerning its similarity with procedures in reduced spaces. It was composed of six structured 5-point Likert scale questions plus two optional open-ended questions. This questionnaire tries to demonstrate level 1 of Kirkpatrick training effectiveness model (reaction) for our new simulator box^17^ ^,^ ^18^. The survey was sent to all participants after assessing the performance of all exercises of the reduced space basic laparoscopic training program (Figure 1). 

### Statistical analysis

A descriptive and analytic statistic was performed. To assess differences between 2 and 3 groups, non-parametric statistic was used, Mann-Whitney and Kruskal Wallis test respectively. All data were recorded on MS Excel® and analyzed using STATA 13. 

## RESULTS

### Reduced space training box

**FIGURE 2:**
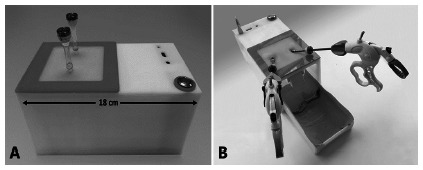
A) Pediatric laparoscopy box; B) sliding tray

Based on the dimensions reported by Azzie et al.^3^ a 10.5 x 10 x 18 cm acrylic simulator was created, with an internal working surface of 9 x 9 cm (Figure 2). A V2 camera was mounted with a Sony^®^ image sensor model IMX2019^®^ of eight fixed megapixels, but with the possibility of making an anteroposterior angle movement from 0 to 180°. The camera is capable to obtain 3280 x 2464 pixels photographs, being also compatible with obtaining videos with 1080p30, 720p60 and 640x480p60/90 resolution. An image processor with HDMI, RCA, and an internal Wi-Fi module was incorporated, which enables streaming and image capture. 

Additionally, it can be programmed to obtain USB output to monitor or Android^®^ mobile. In the upper part of the simulator, a square area with a silicone layer of 9 x 9 cm was placed. This layer imitates the human skin due to its texture and color, and it makes it possible to arrange the trocars for the laparoscopic instruments at different distances to simulate both neonatal procedures and surgical situations in confined spaces in adult patients. Trocars of 3.5 mm were added, which allows working with 3 mm laparoscopic instruments (Figure 2A). Besides, a sliding tray of the same acrylic-washable was designed to contain biological material that enables the development of training models with ex vivo tissue (Figure 2B).

### Reduced Space Basic Laparoscopic Training Program

All exercises that are part of the Basic Laparoscopic Training Program in our center, except for one, were replicated on a scale of 1: 0.5 to compose the Reduced Space Basic Laparoscopic Training Program. The camera-handling exercise was excluded from the analysis because it is performed in a virtual-reality simulator. 

### Construct validity

**FIGURE 3:**
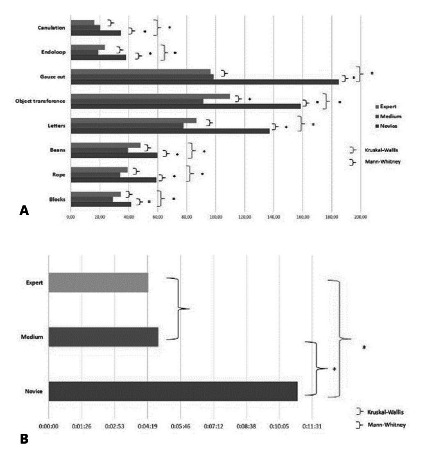
A) Differences in performance considering the expertise; B) differences in the performance of tying a laparoscopic knot

**FIGURE 4:**
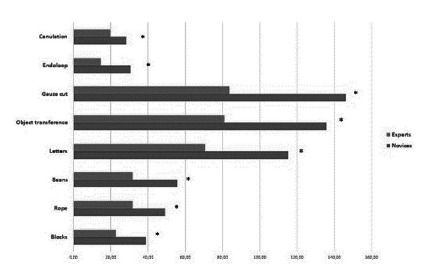
Differences in the performance in “Experts” vs. “Novices”

**FIGURE 5:**
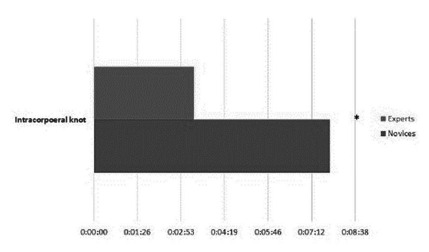
Performance in laparoscopic knot tying

Forty-nine subjects completed the nine exercises. When considering the educational level (surgeon, resident, medical clerk), all tasks showed statistically significant differences upon medical clerks compared to residents and surgeons. Still, only one exercise showed a statistically significant difference between residents and surgeons (Figures 3 A and 3 B). When we compared “Experts” vs. “Novices” all tasks showed a statistically significant difference in performance (Figures 4 and 5).

### Survey to surgeons and residents

**FIGURE 6:**
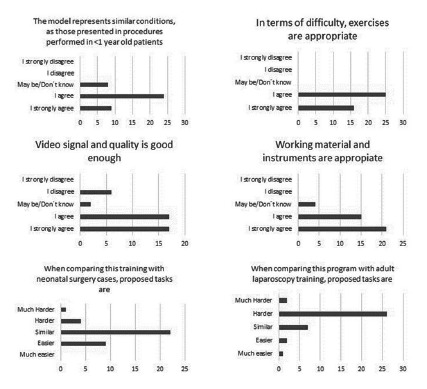
Survey´s results

The survey was answered by 42 of 49 participants, which represents an 85.71%, average age 31.3 years (20-60 years), 42.9% were male, 14 (33.3%) surgeons, 16 (38.1%) surgical residents and 12 (28.6%) surgical clerks. 38.7% (19/42) of the participants reported to perform at least the half of their surgeries laparoscopically, 81% (34/42) of the participants felt that the aspects related to the camera (image quality, angle, zoom) were adequate or very adequate to perform the exercises and 90% (38/42) thought that the work material was sufficient (Figure 6). 81% (34/42) of the participants considered that the simulator reproduces similar conditions to procedures performed in children under one year of age (Figure 6). 62% (26/42) of the participants answered that the simulator represents a difficulty similar to procedures performed in newborns, 25% (10/42) considered that the simulator is easier than a real surgery and 14% (6/42) thought it is more complicated than an actual surgery (Figure 6). When comparing this endotrainer to adult dimensions simulator, 73.7% (31/42) considered that performing the elemental tasks on the neonatal simulator is more complicated, while 18.4% (8/42) responded that the difficulty was similar (Figure 6). When we considered the surgeon’s answers exclusively, we found that all of them (14/14) felt that the simulator reproduces the same conditions to procedures performed in children under one year of age and that the exercises were adequate in difficulty and the working material was sufficient. In contrast, 13/14 considered that the aspects related to the camera were satisfactory.

Finally, concerning if the simulator represents a similar difficulty to procedures performed in newborns: 8/14 thought that the simulator is “very likely” a newborn dimensions, 3/14 considered it “easier,” and 2/14 found it “more difficult.” 

About the optional open-ended questions, were answered by 88% of the participants (37/42) with positive aspects: size, a similarity with actual dimensions and simplicity, were the most frequent comments. On the other hand, the main critics or issues to improved were mentioned by 83% of the participants (35/42): optic view angle and the absence of adjustment in the camera’s focus were the primary critics.

## DISCUSSION

Technological improvement, supporting therapies, and training of surgeons has allowed laparoscopy to become the preferred alternative for many surgical procedures. This progress also brings new challenges, including procedures with extremely delicate structures (e.g., Whipple), in confined spaces (e.g., biliodigestive anastomoses), and neonatal patients (e.g., congenital diaphragmatic hernia). These challenges must be addressed with simulation and trained in the same systematic way as others that have been published^25^ ^,^ ^28^. The laparoscopic simulation training box of reduced spaces allows safe and reproducible training.

In this study, we achieved the goal of developing a laparoscopic training box with a favorable level of reaction by the participants. The new simulator allows not only the training of pediatric surgeons. Still, it may also be useful for surgeons who perform procedures in adult patients under conditions of reduced space, such as hepatobiliary surgery, gynecological, or coloproctological surgery. Additionally, it generates the possibility for training veterinarians in performing procedures on small animals.

Azzie et al.^3^ described a laparoscopic simulator with pediatric dimensions together with the validation of the five exercises described in FLS. This work represented a starting point by designing modifications to the original model to facilitate training and improve the quality of the experience in neonatal laparoscopic simulation. The first step was to adapt our Laparoscopic-Basic-Skills Program to train residents and surgeons in obtaining the basics skills before facing laparoscopic procedures in reduced spaces. Furthermore, our training box allows the use of biological material for the development of ex-vivo models of surgical pathologies through the incorporation of a tray. In this way, the integration of a folding camera with high resolution allows obtaining better image quality for more complex procedures on ex-vivo models without a laparoscopy tower, which also makes this device portable and allows home-use. The silicone layer to simulate the abdominal wall has the advantage of being able to simulate procedures that are performed on the patient’s surface (e.g., ostomies, insertion of auxiliary trocars, altering the disposition of the accesses).

Azzie et al.^3^ calculated the working volume according to the dimensions of the simulation box (1890 ml). However, when building our prototype, it was evident that the working area was smaller, corresponding exclusively to the surface of the platforms (9x9 cm). If the height of the box projects that surface, we obtain a working volume of 729 ml, which could be reduced even more by elevating the tray, reducing the height by half (364.5 ml). Therefore, we consider that this simulator can reproduce surgeries performed in neonates, but these characteristics have yet to be tested in future procedure-based models.

Surgical instruments used in this model (clamps, holder, and 3 mm scissors) are the same as those used in real laparoscopic neonatal surgeries. This simulation training helps to practice precise and delicate handling of structures and for acquiring training with small 3 mm instruments. This issue was the best value by those who used the simulator.

The first step for the training on the new laparoscopic box was the adaptation of each exercise of the Basic Laparoscopic Training Program, based on previously validated programs^10^ ^,^ ^16^ ^,^ ^26^ ^,^ ^27^. The adaptation of these exercises allowed the creation of a Reduced Space Basic Laparoscopic Training Program, for the acquisition of skills in pediatric laparoscopy.

A trainee’s reaction to a new training instance is measured, to assess the different grade of validity is “a must” before a full training program is offered in an institution as the tasks given in FLS and other basic skills training programs do not aim to represent a real scenario but only to train an ability. Face validity, defined as the realism of what is supposed to represent^23^, cannot be fully assessed. Our survey is oriented to determine the similarity concerning work-volume between the simulator and pediatric/neonatal procedures, but it cannot be taken as face validity. Another level of validation is construct validity^23^, which aims to determine if the proposed task and simulator can successfully discriminate different levels of expertise. We defined an expert as the one who has already received simulated laparoscopic training and performed most of his procedures laparoscopically. Under these conditions, our program successfully reached construct validity on all its exercises. We did that analysis considering that most of our residents have previous training and the fact that each condition on its own could not explain a good performance. This step also allows us to define time objectives on each task, and the ten lower time percentile seems to be a good cutoff in our context. To determine time goals for each task was not an objective in this study, then no further analysis was made. 

An essential limitation of this type of study is the definition of “expert” subjects for whom there is no universal consensus, and frequently this definition is based on the local context on which the protocol is performed. On the other hand, when assessing essentials laparoscopic skills, exposure to previous simulated training programs could significantly diminish performance differences among groups (residents and staff surgeons). However, some evidence shows that the reduction of surgical space is a determining factor in the performance of experts^14^. Nevertheless, the analysis of data in a population of residents who regularly receive training compared to a group of experts in which exposure to simulated training programs is not universal could be the explanation for the little difference found when performance was analyzed addressed by academic level. We think that this difference will probably grow when validating advanced procedure-based models in the future.

Another aspect of being considered is the measurement of time as the only factor by which exercises were evaluated. Other procedure assessment tools include manipulation of material outside the visual field, objective measure of surgical skills (OSATS), total distance covered by the instruments or hands, etc. We decided to use the execution time of the exercise for its simplicity and objectivity, knowing that the time recorded in this study was only taken in count whenever the training was realized without faults. However, we identified that there are two exercises in which some form of surgical technique evaluation is required; these exercises are gauze cutting and knot making.

## CONCLUSION

The simulator has a high-quality image that allows the practice with a smaller size modified basic exercises but also with ex-vivo models. The endotrainer was successfully evaluated by laparoscopic pediatric surgeons and surgery residents, and it discriminates between different levels of expertise. It represents a new alternative to acquiring basic laparoscopic skills in reduced space and represents a standardized box in which develop simulation models of neonatal procedures.
